# Statistical air quality predictions for public health surveillance: evaluation and generation of county level metrics of PM_2.5_ for the environmental public health tracking network

**DOI:** 10.1186/1476-072X-12-12

**Published:** 2013-03-14

**Authors:** Ambarish Vaidyanathan, William Fred Dimmick, Scott R Kegler, Judith R Qualters

**Affiliations:** 1 Centers for Disease Control and Prevention, National Center for Environmental Health, Mail Stop: F60; 4770 Buford Hwy, Atlanta, GA, 30341, USA; 2 Office of Research and Development, US Environmental Protection Agency, Mail Stop: D343-04, 109 Alexander Drive, Durham, NC, 27711, USA; 3 Centers for Disease Control and Prevention, National Center for Injury Prevention and Control, Mail Stop: F64; 4770 Buford Hwy, Atlanta, GA, 30341, USA

**Keywords:** Particulate matter, Tracking Network, Hierarchical Bayesian, Air quality system, Geo-imputation

## Abstract

**Background:**

The Centers for Disease Control and Prevention (CDC) developed county level metrics for the Environmental Public Health Tracking Network (Tracking Network) to characterize potential population exposure to airborne particles with an aerodynamic diameter of 2.5 μm or less (PM_2.5_). These metrics are based on Federal Reference Method (FRM) air monitor data in the Environmental Protection Agency (EPA) Air Quality System (AQS); however, monitor data are limited in space and time. In order to understand air quality in all areas and on days without monitor data, the CDC collaborated with the EPA in the development of hierarchical Bayesian (HB) based predictions of PM_2.5_ concentrations. This paper describes the generation and evaluation of HB-based county level estimates of PM_2.5_.

**Methods:**

We used three geo-imputation approaches to convert grid-level predictions to county level estimates. We used Pearson (**r**) and Kendall Tau-B (**τ**) correlation coefficients to assess the consistency of the relationship, and examined the direct differences (by county) between HB-based estimates and AQS-based concentrations at the daily level. We further compared the annual averages using Tukey mean-difference plots.

**Results:**

During the year 2005, fewer than 20% of the counties in the conterminous United States (U.S.) had PM_2.5_ monitoring and 32% of the conterminous U.S. population resided in counties with no AQS monitors. County level estimates resulting from population-weighted centroid containment approach were correlated more strongly with monitor-based concentrations (**r** = 0.9; **τ** = 0.8) than were estimates from other geo-imputation approaches. The median daily difference was −0.2 μg/m^3^ with an interquartile range (IQR) of 1.9 μg/m^3^ and the median relative daily difference was −2.2% with an IQR of 17.2%. Under-prediction was more prevalent at higher concentrations and for counties in the western U.S.

**Conclusions:**

While the relationship between county level HB-based estimates and AQS-based concentrations is generally good, there are clear variations in the strength of this relationship for different regions of the U.S. and at various concentrations of PM_2.5_. This evaluation suggests that population-weighted county centroid containment method is an appropriate geo-imputation approach, and using the HB-based PM_2.5_ estimates to augment gaps in AQS data provides a more spatially and temporally consistent basis for calculating the metrics deployed on the Tracking Network.

## Background

Numerous studies have identified a relationship between fine particulate air pollution and its impact on human health [1]. Particles with an aerodynamic diameter of 2.5 μm or less (PM_2.5_) are small enough to invade air pathways in the body, and have been known to cause adverse health effects [2]. Several epidemiologic and human clinical studies have examined the cardiovascular and respiratory health effects of both acute and long term exposures to PM_2.5_[3-5]. The Medicare Air Pollution Study (MCAPS), a multi-city study in the United States (U.S.), reported a short-term increase in hospital admission rates associated with elevated ambient PM_2.5_ concentrations, for health outcomes such as ischemic heart disease, heart failure, chronic obstructive pulmonary disease and respiratory infection [6]. The MCAPS study also concluded that the cardiovascular risks, estimated at the county level, tended to be higher in the eastern U.S. Similarly, the extended follow-up of the Harvard Six Cities study showed that cardiovascular and lung cancer mortality were positively associated with long-term ambient concentrations of PM_2.5_[7].

To quantify the health impacts of PM_2.5_, and to track population exposure to PM_2.5_, accurate and timely data collected on an ongoing basis are needed at the sub-state level. The Pew Environmental Health Commission report released in 2000 found that the state of environmental public health systems were fragmented and not robust enough to respond to environmental threats [8]. Based on the recommendations of the Pew Commission, the U.S. Congress funded the Centers for Disease Control and Prevention (CDC) to establish a National Environmental Public Health Tracking Program. The cornerstone of this program is the National Environmental Public Health Tracking Network (Tracking Network) which provides nationally consistent data and metrics (indicators and measures) to monitor relationships among hazards, exposures, and health effects [9]. The CDC, U.S. Environmental Protection Agency (EPA), and state local health departments funded by the CDC have been collaborating in the development of air quality metrics for PM_2.5_ for integration into the Tracking Network (http://ephtracking.cdc.gov/showAirData.action). In July 2009 during the initial launch of the Tracking Network, only Federal Reference Method (FRM) Air Quality System (AQS) monitor data were incorporated into the Network to provide county level air quality metrics.

**Figure 1:**
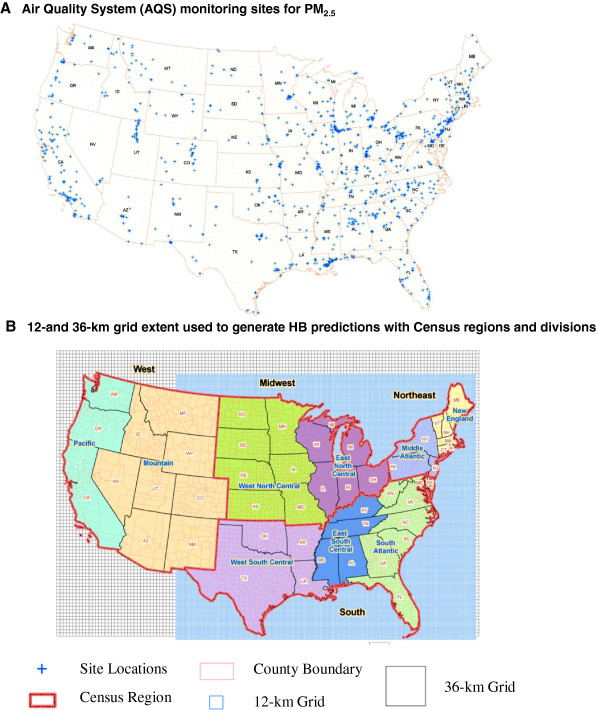
**Spatial coverage of monitoring and modeled air quality data.** “Blue cross sign” = Site Locations; “dotted rectangular red” = Census Region: “pink rectangle = County Boundary: “smaller blue square = 12-km Grid: big gray square = 36-km Grid.

While AQS monitor data are viewed as the “gold standard” for characterizing ambient air quality and determining compliance with National Ambient Air Quality Standards (NAAQS), such data are limited in space and time (Figure 1A) [10,11]. During the year 2005, fewer than 20% of counties in the conterminous U.S. (leaving out 32% of the resident population) were monitored for PM_2.5_ and most monitors operated every third day [12]. As a result, the AQS-based metrics on the Tracking Network are adjusted to account for missing days in monitor data (http://ephtracking.cdc.gov/showIndicatorPages.action). Also, when AQS data are available from multiple monitors for a given county and day, the highest 24-h average (daily) concentration among all the monitors is selected for purposes of calculating the measures. These adjustments are consistent with EPA practices and were adopted by the Tracking Network to ensure that the AQS-based metrics for PM_2.5_ do not understate the air quality problem in any given area [13].

In order to better understand air quality for areas and days without monitor data, the CDC collaborated with the EPA on the development of a hierarchical Bayesian (HB) model to predict daily PM_2.5_ concentrations for use in the Tracking Network. The HB model integrates AQS monitor data with results from the EPA’s Community Multiscale Air Quality (CMAQ) model to generate predicted PM_2.5_ concentrations for a 36-km grid (individual predictions for 36-km × 36-km grid cells) across the conterminous U.S. and for a 12-km grid across the eastern half of the U.S. (http://www.cmaq-model.org). The statistical approach incorporates prior knowledge of the model parameters in the hierarchical Bayesian model, which results in improved estimation of the PM_2.5_ concentrations in areas (also covered by the CMAQ grid) and at times (days) that are not monitored [14]. The model also quantifies the prediction error associated with the predicted daily concentrations for each grid cell. In general, the HB model utilizes monitor data, and bias-adjusted CMAQ model output for non-monitored areas and days. Background documents for the HB model can be found on the Tracking Network and at the EPA webpage (http://www.epa.gov/heasd/sources/projects/CDC/index.html).

The remainder of this paper describes the utility of HB predictions for public health surveillance purposes and their use in the development of county level PM_2.5_ metrics for the Tracking Network. Using monitor and model data for the year 2005, we considered the following:

1. creating a spatial relationship between grid cells and counties so that daily county level estimates can be generated from HB predictions;

2. evaluating whether HB predictions at the 12- or 36-km resolution PM_2.5_ should be used for the calculation of daily county level estimates;

3. comparing the resultant daily county level HB estimates with AQS county level monitor data;

4. comparing county level annual averages of PM_2.5_ based on HB estimates with those based on AQS monitor data.

## Methods

### Geo-imputation methods

**Figure 2:**
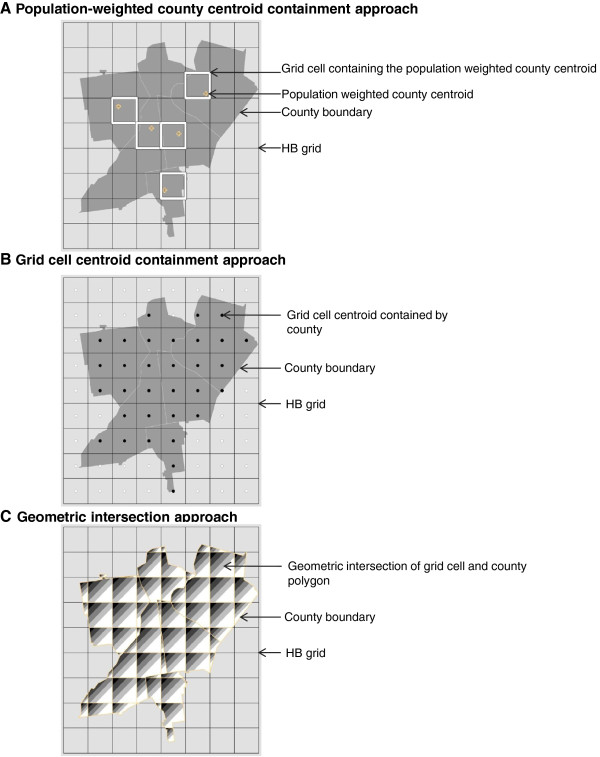
Geo-imputation methods.

Figure 1B shows the spatial extent of the 12- and 36-km grids with respect to counties in the conterminous U.S. In order to relate grid cells to comparatively irregular county geography and assign daily HB grid-level predictions of PM_2.5_ to counties, geo-imputation methods were employed [15,16]. For both grid resolutions, we compared three different geo-imputation methods. First, we examined a population-weighted county centroid containment approach [17,18]. This approach involves two steps. For a given county indexed by k, we first determined the population-weighted county centroid) *(C(X*_*k*_*,Y*_*k*_*))* by calculating the spatial mean center across the geometric centroids of all census blocks covered by the county, using the population fraction of each census block as the assigned weight:

$$X_{k}=\sum_{j=1}^{n_{k}}X_{B_{j,k}}\times \frac{P_{B_{j,k}}}{\sum_{j=1}^{n_{k}}P_{B_{j,k}}}$$

$$Y_{k}=\sum_{j=1}^{n_{k}}Y_{B_{j,k}}\times \frac{P_{B_{j,k}}}{\sum_{j=1}^{n_{k}}P_{B_{j,k}}}$$

where:

*n*_*k*_ = number of census blocks in county k;

*X*_*Bj,k*_ = X coordinate of the centroid of census block j contained within county k;

*Y*_*Bj,k*_ = Y coordinate of the centroid of census block j contained within county k;

*P*_*Bj,k*_ = population of census block j contained within county k;

We next related each population-weighted county centroid to the grid cell into which it falls. Based on this determination (Figure 2A), we assigned the daily concentration values of grid cells to counties. This produced daily county level estimates

$$\left(C_{\mathrm{ES}T_{i,k}}\right)$$

Of PM_2.5_ for counties in the conterminous U.S. based on HB predictions, where i denotes the day and k denotes the county.

Our second approach was again based on centroid containment and relates all grid cell centroids (geometric) to the county into which they fall [19]. We established a relationship between each given county boundary polygon and all the grid cell geometric centroids it contains, and then transferred HB predictions to that county (Figure 2B). For counties that did not contain a grid cell geometric centroid, which were very few, we related the nearest one. Since many counties contain more than one grid cell centroid, we selected the maximum predicted concentration value for each day from all the grid cells with centroids in each given county to create daily county level estimates of PM_2.5_. This is consistent with the EPA approach of using the maximum concentration among multiple monitors in a county.

The third approach was based on geometric intersection of the county boundary and grid cell polygons [20]. We performed an intersect-overlay analysis to identify geometric intersections between grid cells and counties and related each county to grid cells that either fell within or intersected with the county boundary (Figure 2C). After establishing the appropriate many-to-many relationship between counties and grid cells, we selected the maximum HB prediction for each day from all the grid cells related to each given county to create daily county level estimates of PM_2.5_ from HB predictions.

### Evaluation of county level estimates of PM_2.5_

We compared the county level PM_2.5_ estimates derived from predictions at the 12- and 36-km resolutions with AQS-based concentrations in order to assess the quality of the model outputs as well as the effects of grid resolution. We used Pearson (**r**) and Kendall Tau-B (**τ**) correlation coefficients to assess the consistency of the relationship between the HB- and AQS-based PM_2.5_ concentrations [21,22]. For a set of daily data points (AQS_1_, HB_1_), (AQS_2_, HB_2_), …, (AQS_n_, HB_n_) **r** is calculated as:

$$r=\frac{\sum_{i=1}^{n}\left(\mathrm{AQ}S_{i}-\bar{\mathrm{AQS}}\right)\left(HB_{i}-\bar{\mathrm{HB}}\right)}{\sqrt{\sum_{i=1}^{n}{\left(\mathrm{AQ}S_{i}-\bar{\mathrm{AQS}}\right)}^{2}}\sqrt{\sum_{i=1}^{n}{\left(HB_{i}-\bar{\mathrm{HB}}\right)}^{2}}}$$

where

$$\bar{\mathrm{AQS}}$$

and

$$\bar{\mathrm{HB}}$$

represent averages across the n data points.

To calculate **τ,** n(n-1)/2 pairs of data points are classified as concordant or discordant. A concordant pair is any pair for which the ranks of AQS and HB agree, i.e., for any pair of observations (AQS_p_, HB_p_) and (AQS_q_ HB_q_), both AQS_p_ > AQS_q_ and HB_p_ > HB_q_ or both AQS_p_ < AQS_q_ and HB_p_ < HB_q_. A discordant pair is any pair of observations for which the ranks for AQS and HB disagree, i.e., either AQS_p_ > AQS_q_ and HB_p_ < HB_q_ or AQS_p_ < AQS_q_ and HB_p_ > HB_q_[23]. With C and D respectively denoting the number of concordant and discordant pairs (assuming no ties), the value of **τ** is then calculated as:

$$\tau =\frac{C-D}{n\left(n-1\right)/2};$$

the denominator is adjusted accordingly in the event of ties [24].

We compared the county level PM_2.5_ estimates derived from HB predictions with the county level AQS-based PM_2.5_ concentrations at the daily and annual levels. The EPA provided the daily county level AQS data product for PM_2.5_ by selecting the daily maximum monitor value of all FRM monitors operating in a given county. We examined the consistency of the relationship (**r** and **τ**), median difference (AD) and median relative difference (RD) between daily county level estimates of PM_2.5_ generated from HB predictions and AQS-based concentrations. The AD and RD for a given county k with n daily data points are defined as:

$$\mathrm{ADk}=\mathrm{median}\;\left\{\left(C_{\mathrm{ES}T_{1,k}}-C_{\mathrm{AQ}S_{1,k}}\right),\ldots ,\left(C_{\mathrm{ES}T_{n,k}}-C_{\mathrm{AQ}S_{n,k}}\right)\right\}$$

$$RD_{k}=\mathrm{median}\left\{\frac{\left(C_{\mathrm{ES}T_{1,k}}-C_{\mathrm{AQ}S_{1,k}}\right)}{C_{\mathrm{AQ}S_{1,k}}}\times 100,\ldots ,\frac{\left(C_{\mathrm{ES}T_{n,k}}-C_{\mathrm{AQ}S_{n,k}}\right)}{C_{\mathrm{AQ}S_{n,k}}}\times 100\right\}$$

where:

$$C_{\mathrm{ES}T_{i,k}}$$

=HB-based estimate for day i and county k;

$$C_{\mathrm{AQ}S_{i,k}}$$

= AQS-based concentration for day i and county k.

We further calculated PM_2.5_ annual averages using the daily county level estimates of PM_2.5_ generated from the HB-based predictions. We created two forms of this annual-level measure. One form used county level estimates from HB predictions only; the other form used the county level estimates from HB predictions for counties and days without monitor data and AQS data for counties and days with monitor data. We compared these annual averages to annual averages derived exclusively from AQS data (as were available initially on the Tracking Network) using Tukey mean-difference plots; such plots are primarily used for identifying the presence of fractional bias. A Tukey mean-difference plot is a scatter plot with *(X*_*k*_*,Y*_*k*_*)* points defined as:

$$X_{k}=\frac{\left({\bar{HB_{k}}}^{*}+\bar{\mathrm{AQ}S_{k}}\right)}{2}$$

$$Y_{k}={\bar{HB_{k}}}^{*}-\bar{\mathrm{AQ}S_{k}}$$

where:

$$\bar{\mathrm{AQ}S_{k}}$$

= AQS-based annual average for county k;

HBk¯*

= annual average for county k from HB-based estimates only; or alternatively from the combination of HB-based estimates and AQS-based concentrations.

We carried out our data analyses using the Statistical Analysis System (SAS® Version 9.2) and Environmental Systems Research Institute’s GIS software (ESRI, ArcGIS® Version 9.3). This study was determined to be research not involving human subjects by the CDC National Center for Environmental Health (NCEH) Office of Science. This study did not require further review by the CDC institutional review board.

## Results

For 2005, 587 (19%) of counties in the conterminous U.S. had PM_2.5_ monitors that operated year-round. Most of these PM_2.5_ monitors only operated every third day while some operated every sixth day. A few monitors operated on an every-day schedule. HB predictions available at the 36-km grid resolution were available for 11266 grid cells covering the entire conterminous U.S. It should be noted that CMAQ estimates dominated the 36-km HB predictions in the western areas where few monitors are located. The 12-km HB predictions were available for the eastern U.S. with 66960 grid cells, out of which 66123 grid cells overlapped and were aligned with the 36-km grid.

### Comparison of Geo-imputation methods

We applied the geo-imputation procedures to both 12- and 36-km grid-level predictions to generate daily county level PM_2.5_ estimates. Table 1 shows the correlations between daily county level HB estimates of PM_2.5_, derived using the three different geo-imputation methods, and daily county level AQS-based PM_2.5_ concentrations. We carried out the analysis for all counties and days with monitor data. The population-weighted county centroid containment approach used to convert 36-km grid-level predictions to county level estimates performed slightly better (r = 0.94, τ = 0.83) than other two geo-imputation approaches considered in this assessment. We selected these estimates for all subsequent analyses. Additionally, the county level estimates of PM_2.5_ derived from 36-km HB predictions performed better than the estimates derived from 12-km HB predictions for all the geo-imputation approaches.

**Table 1:** **Correlation between county level HB estimates of PM**_**2**.**5**_**and AQS**-**based PM**_**2**.**5**_**concentrations**

**Geo**-**imputation approach**	**Grid resolution**	**Correlation coefficient**	
		**Pearson(r)**	**Kendall Tau**-**B(τ)**
Population-weighted county centroid containment	36-km	0.94	0.83
	12-km	0.86	0.69
Grid cell centroid containment	36-km	0.92	0.78
	12-km	0.84	0.67
Geometric intersection	36-km	0.91	0.76
	12-km	0.84	0.66

### Daily county level comparison

**Figure 3:**
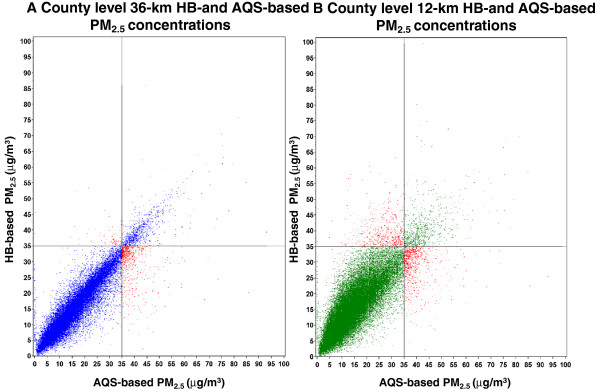
**Daily comparison of county level estimates of HB- and AQS**-**based PM**_**2**.**5**_**concentrations.**

The daily county level PM_2.5_ concentration estimates derived from 12- and 36-km HB predictions were highly correlated with county level AQS-based PM_2.5_ concentrations, with estimates derived from 36-km predictions showing relatively less deviation than estimates derived from 12-km predictions (Figures 3A and 3B). The reference lines in the figures indicate the daily NAAQS for PM_2.5_, which is set at 35 μg/m^3^. The red points in the upper left quadrant indicate county level HB-based estimates above the NAAQS with corresponding AQS-based concentrations below the NAAQS (over prediction near the NAAQS). The red points in the lower right quadrant indicate county level HB estimates below the NAAQS with corresponding AQS-based concentrations above the NAAQS (under prediction near the NAAQS). Overall the figures indicate that for concentrations near the NAAQS, under prediction is more common than over prediction. We proceeded with selecting the population-weighted county level estimates derived from 36-km HB predictions for creating county level metrics and for further analysis. The median daily difference (by county) between daily county level HB-based estimates derived from 36-km predictions and AQS-based concentrations was −0.2 μg/m^3^ with an interquartile range (IQR) of 1.9 μg/m^3^ and the median relative daily difference (by county) was −2.2% with an IQR of 17.2%.

In order to understand further the issue of under prediction and over prediction at different levels of PM_2.5_ and across various parts of the U.S., we evaluated the daily county level differences for different AQS-based concentration range and census division. Table 2 shows the median AD and RD stratified by these factors. We established the concentration ranges by dividing the AQS-based concentrations into two groups, those greater than 35 μg/m^3^ and those less than or equal to 35 μg/m^3^. We further subdivided the group consisting of concentrations less than or equal to 35 μg/m^3^ into three groups using a tertile classification scheme.

**Table 2:** **Daily differences between county level HB estimates of PM**_**2**.**5**_**and AQS**-**based PM**_**2**.**5**_**concentrations by census regions and divisions**

**Census region**	**Census division**	**AQS-based PM**_**2.5**_**concentration ranges**							
		**(0**–**8)μg/m**^**3**^		**(9–14)μg/m**^**3**^		**(15–35)μg/m**^**3**^		**(>35) μg/m**^**3**^	
		**Median absolute difference(μg/m**^**3**^) *	**Median relative difference(%)**^**α**^	**Median absolute difference(μg/m**^**3**^)*	**Median relative difference(%)**^**α**^	**Median absolute difference(μg/m**^**3**^)*	**Median relative difference(%)**^**α**^	**Median absolute difference(μg/m**^**3**^)*	**Median relative difference(%) **^**α**^
Midwest	East North Central	0.2	4.8	0.0	−0.4	−1.0	−4.6	−3.4	−8.4
		(−0.2 – 0.8)	(−3.9 – 16.0)	(−0.8 – 0.7)	(−7.2 – 6.7)	(−2.4 – 0.2)	(−11 – 1.0)	(−6.5 – -1.5)	(−14.5 – -3.6)
	West North Central	0.2	3.5	−0.3	−3.3	−1.3	−6.7	−3.3	−7.5
		(−0.3 – 0.6)	(−6.2 – 13.9)	(−1.2 – 0.4)	(−11.0 – 3.6)	(−2.7 – -0.2)	(−13 – -1.1)	(−4.9 – -1.6)	(−12.9 – -3.9)
Northeast	Middle Atlantic	0.3	6.8	0.0	0.0	−1.1	−5.1	−5.3	−12.2
		(−0.2 – 1)	(−3.7 – 20.1)	(−1 – 0.9)	(−8.9 – 8.3)	(−2.9 – 0.4)	(−13.6 – 1.8)	(−12.3 – -2.3)	(−28.3 – -6.1)
	New England	0.1	1.4	−0.6	−5.2	−1.6	−7.9	−3.7	−10.2
		(−0.5 – 0.6)	(−9.7 – 14.1)	(−1.5 – 0.3)	(−14.6 – 3.1)	(−3.4 – -0.1)	(−16.6 – -0.6)	(−7.9 – -2.3)	(−19.6 – -6.1)
South	East South Central	0.3	5.8	0.0	0.3	−0.8	−4.0	−3.2	−7.7
		(−0.2 – 1)	(−3.1 – 17.4)	(−0.7 – 0.8)	(−6.3 – 7.2)	(−2.0 – 0.3)	(−9.8 – 1.6)	(−6.6 – -1.3)	(−15.4 – -3.2)
	South Atlantic	0.1	2.0	0.0	−0.3	−0.7	−3.3	−2.9	−7.5
		(−0.4 – 0.7)	(−7.4 – 12.1)	(−0.8 – 0.7)	(−7.7 – 7.0)	(−1.9 – 0.4)	(−9.5 – 2.4)	(−5.5 – -1.1)	(−14.4 – -2.9)
	West South Central	0.1	2.2	−0.3	−2.9	−1.0	−5.0	−4.7	−12.5
		(−0.4 – 0.7)	(−5.8 – 12)	(−1.1 – 0.5)	(−10.1 – 4.3)	(−2.4 – 0.1)	(−12.1 – 0.6)	(−9.4 – -2.1)	(−24.1 – -5.3)
West	Mountain	0.1	1.7	−1.4	−13.7	−4.4	−23.3	−15.2	−35.3
		(−0.5 – 0.7)	(−9.5 – 17.7)	(−2.7 – -0.3)	(−25.8 – -3.5)	(−8.5 – -1.9)	(−42.4 – -9.9)	(−25.2 – -8.2)	(−55.7 – -16.2)
	Pacific	0.1	2.9	−0.6	−5.9	−2.9	−14.1	−10.9	−22.3
		(−0.5 – 0.8)	(−9.6 – 17.6)	(−1.7 – 0.5)	(−15.8 – 4.5)	(−5.9 – -0.8)	(−28.9 – -4.0)	(−20.7 – -5.8)	(−43.1 – -12.5)

*. Median difference with interquartile range.

^**α**^ Median relative difference with interquartile range.

The HB estimates comported well with AQS data when AQS-based concentrations were less than 35 μg/m^3^; the median AD and median RD ranged from −4.4 to +0.3 μg/m^3^ and from −23.3 to +6.8%, respectively. For concentrations less than or equal to 35 μg/m^3^, the median AD for the Midwest, Northeast, and South ranged from −1.3 to +0.2, -1.6 to +0.3, and −1.0 to +0.3 μg/m^3^, respectively; the West had a median AD range of −4.4 to +0.1 μg/m^3^, notably wider and more indicative of bias than the ranges observed for other census regions. When the prevailing AQS-based concentrations were greater than 35 μg/m^3^, county level HB-based estimates under predicted AQS-based concentrations across all census regions and divisions. For concentrations greater than 35 μg/m^3^, the median AD ranged between −15.2 and −3.2 μg/m^3^_,_ and the median RD ranged between −35.3% and −7.5%. Counties in the Mountain and Pacific census divisions in the western U.S. showed the highest magnitude of under prediction.

### Comparison of annual averages

**Figure 4:**
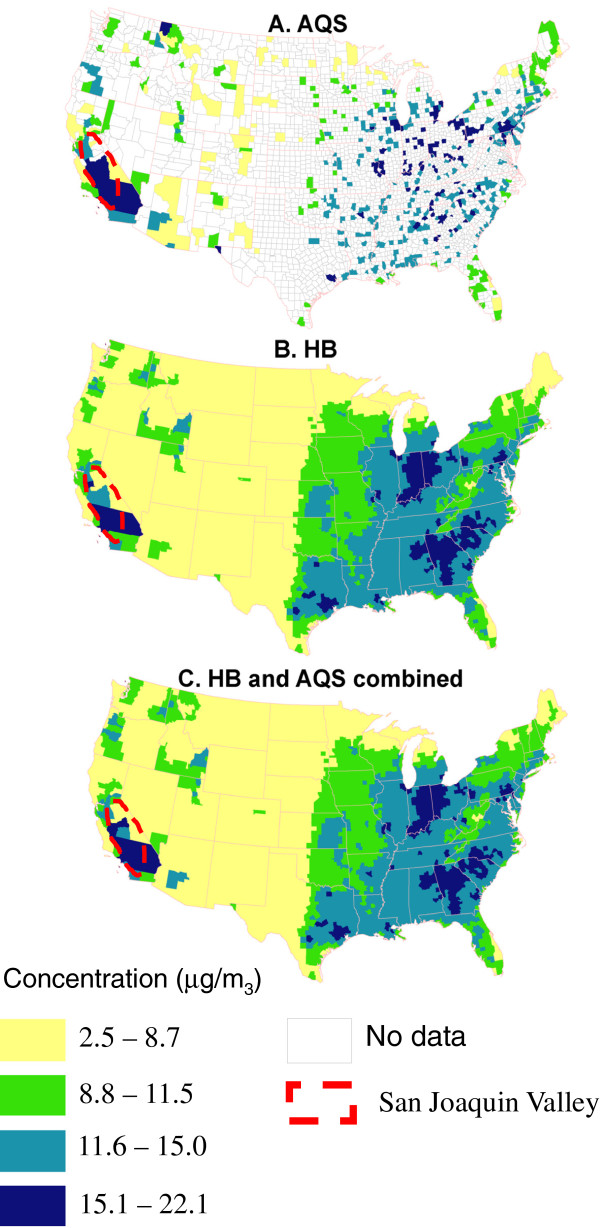
**PM**_**2**.**5**_**annual average concentration by counties.** Concentration (μg/m^3^). “Yellow rectangle = 2.5-8.7: “green rectangle” = 8.8-11.5: “light blue rectangle” = 11.6-15.0: “purple rectangle” = 15.1-22.1: “white rectangle” = No data: “red broken line” = San Joaquin Valley.

**Figure 5:**
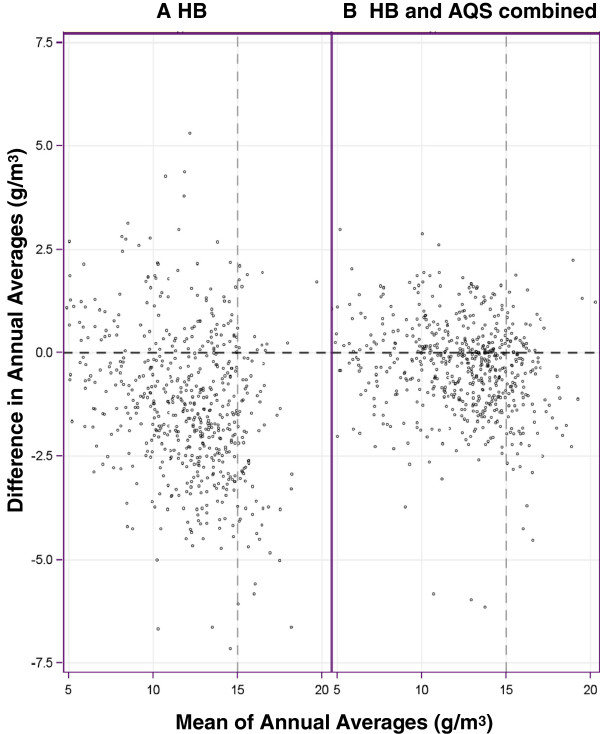
**Tukey mean**-**difference plot of annual averages.**

Figures 4A, 4B, and 4C show county level annual averages calculated using three different approaches. Figure 4A shows annual average PM_2.5_ concentrations based on AQS estimates only. Figures 4B and 4C show two ways to use the HB-based estimates to derive county level annual average concentrations: HB exclusively and HB substituted only for locations and days with missing data, respectively. In Figures 4B and 4C, annual averages were available for all counties in the conterminous U.S. In comparing Figures 4A and 4B, it was evident that in certain areas of the U.S., the under estimation of the HB-based estimates reduced the apparent extent of elevated PM_2.5_. For example, in San Joaquin valley of California, HB-based annual averages for certain counties were lower than the averages derived from AQS data. In Figure 4C, the comparison for San Joaquin Valley is more favorable than the comparison involving Figure 4B, with more counties in the valley having annual average concentrations closer to the averages derived exclusively from AQS data. Tukey mean-difference plots (Figure 5A and 5B) show that using annual averages from HB-based estimates exclusively resulted in under prediction at higher concentrations, while substituting HB-based predictions only for missing counties and days produced less dispersion of values near the zero difference line and lowered the magnitude of differences at higher annual average concentrations, especially near the annual NAAQS.

## Discussion

Spatio-temporal gaps in monitoring can result in uncertainty in the ascertainment of population-level exposures, especially when fluctuations in PM_2.5_ concentrations tend to occur at frequencies not detectable given existing monitor sampling schedules. Monitors that operate for regulatory purposes are not usually sited very close to sources, where high concentrations of PM_2.5_ can be observed. Rather they are sited in places to measure levels of PM_2.5_ that represent average concentration levels over large areas. Lack of PM_2.5_ concentration measurements over continuous spatial and temporal scales limits our ability to link air quality levels with health effects data. Thus, modeled predictions may provide a suitable alternative for use in public health surveillance.

CMAQ model output as well as output from any model that relies on CMAQ output has a spatial unit, the grid cell that differs from the spatial unit of health and demographic data, which are often available at geo-political resolutions, such as county, census tract, etc. Geo-imputation procedures are necessary to convert grid-level data to county level estimates, which are needed to generate metrics for environmental public health surveillance through the CDC Tracking Network and for linkage to spatially comparable health data. The three geo-imputation methods mentioned in this paper are routinely used in public health and other allied fields. In our study, a population-weighted county centroid containment approach performed best among the methods considered for translating grid-level HB predictions to county level estimates. Also, a population-weighted county centroid denotes a spatial mean of the underlying population distribution within each county and estimates of PM_2.5_ generated using this method are intended to represent the ambient concentration levels to which most of the population are potentially exposed.

In the context of linking with health data, the spatial scale of modeled predictions was a very important consideration in developing county level PM_2.5_ estimates. For 2005, HB predictions of PM_2.5_ were available at a 12-km resolution for the eastern U.S., whereas HB predictions of PM_2.5_ were available at a 36-km resolution for the entire conterminous U.S. County level estimates of PM_2.5_ derived from 36-km predictions correlate more strongly with AQS-based PM_2.5_ concentrations than do estimates derived from 12-km predictions. The predominant reason for the difference in performance of 12- and 36-km HB predictions was the underlying CMAQ estimates. The input needed to generate the 12-km and 36-km CMAQ estimates were processed with different assumptions and, for certain inputs, resolving to a finer spatial scale could add uncertainty to the final model output [25]. We developed county level estimates of PM_2.5_ from 36-km HB predictions since our primary goals were to have the HB-based metrics approach the values of the AQS-based metrics, and generate metrics for the entire conterminous U.S.

The strength and consistency of the relationship between daily HB- and AQS-based PM_2.5_ county level estimates are acceptable at concentrations below the daily NAAQS and, at these concentrations, differences between HB- and AQS-based PM_2.5_ estimates are reasonable for most census regions. For 2005, less than 2% of the measurements available from AQS monitoring reflected concentrations greater than 35 μg/m^3^. At these higher concentrations, HB-based county level estimates are more likely to under predict AQS concentrations, with the largest differences observed for the western region of the U.S. Some of these differences can be explained based on model features. The HB model fuses monitor data when available with CMAQ estimates and, for most locations, days with only CMAQ estimates outnumber days with both CMAQ and AQS data. CMAQ estimates are primarily used for predicting background concentrations and do not adequately capture spikes in air quality levels as a result of exceptional events [26]. While the bias in the CMAQ estimates is adjusted using a global (national-level) regression approach, and the AQS data measurement error is accounted for in the HB model , the daily HB predictions can be different from the coincident AQS measurements when CMAQ estimates greatly differ from the AQS data. Additionally, there are relatively fewer monitor-based observations available for the western U.S. and CMAQ estimates under predict AQS concentrations in the western U.S., especially when the terrain is mountainous [27]. Hence, HB estimates rely heavily on CMAQ in the western U.S. and we see larger absolute and relative differences between county level HB and AQS estimates with increasing PM_2.5_ concentrations (Table 2). Users of HB-based PM_2.5_ estimates should be aware of the limitations of these data as well as the benefits of having data over continuous spatio-temporal scales.

Annual county level metrics of PM_2.5_, such as annual averages, provide a useful summary of prevailing concentration levels. However, averages created from AQS-based PM_2.5_ concentration measurements are limited to counties with monitors and therefore do not provide a complete picture of prevailing air quality throughout the conterminous U.S. Moreover, PM_2.5_ annual metrics derived from AQS data based on a sampling frequency that is predominantly every third day can be taken to accurately characterize general conditions only under the assumption that days included in the sample fairly represent the full calendar. Given that the HB-based estimates are available for every day of the year, annual averages incorporating these estimates can be interpreted without any assumptions concerning days without data.

The benefits of employing HB predictions should be considered in light of the added uncertainty which they introduce. As noted, the annual county level HB-based annual averages can understate or overstate the air quality problem in specific areas compared to averages based on AQS concentrations. At higher concentrations, especially near the annual NAAQS—15 μg/m^3^, and in the western U.S., HB-based annual averages are more likely to fall below monitor-based measurements. Notably, combining HB-based estimates with AQS-based concentrations results in annual averages that comport well with annual averages created using AQS data exclusively; however, a few counties have lower annual averages when compared with the annual averages obtained exclusively from AQS-based concentrations.

In summary, we characterized the difference between HB-based estimates and AQS-based concentrations with the intent that the results can guide public health professionals and researchers on the appropriate use of the county level estimates of PM_2.5_. Our analysis of daily differences between AQS-based concentrations and HB-based estimates of PM_2.5_ indicate that the differences can vary across census regions and divisions, and that generally HB-based county level estimates tend to under predict at higher concentrations. This needs to be considered when using daily county level HB-based estimates to identify exceedances of the daily NAAQS in different parts of the country or to conduct studies that assess health outcomes related to short-term PM_2.5_ exposures. The annual averages developed by combining HB- and AQS-based PM_2.5_ data show less variation with AQS-based annual averages. Given the need to correctly characterize air quality levels and minimize the discrepancy with county level annual averages created from AQS data that are commonly used, we suggest that the county level annual averages of PM_2.5_ for the Tracking Network be calculated using AQS data when and where they are available and using HB-based estimates for days and locations without such data.

## Conclusions

Poor air quality is a worldwide problem. The global burden of disease report (2010) identifies air pollution as a major contributor to premature mortality [28]. Measurements from monitors are limited in space and time, and modeled data can be an alternative to ascertain population level exposures. Our evaluation of HB predictions and AQS measurements explores the utility of modeled data from a public health perspective. Using the HB-based predictions to augment gaps in AQS data provides a more spatially and temporally consistent basis for calculating the metrics deployed on the Tracking Network. Further, “data fusion” techniques, combining monitor and modeled data, are being used in many countries to produce grid-level air quality predictions [29]. The evaluation framework presented in this paper is robust and can be extended to areas outside the U.S.

Converting grid-level predictions to estimates by geo-political units, such as counties or county equivalents, is needed to link health and population information with air pollutant data. This manuscript suggests that assigning modeled predictions to counties using a population-weighted centroid containment method is an effective approach for making the translation between a fixed grid and county-specific geography. Counties or similar administrative units exist in several European counties, for example, and the geo-imputation methods suggested in the paper can be adopted seamlessly to obtain pollutant concentrations to which most of the population is potentially exposed.

## Abbreviations

AD: Absolute difference; AQ: Air quality; AQS: Air quality system; CDC: Centers for disease control and prevention; CMAQ: Community multi-scale air quality; EPA: US environmental protection agency; ESRI: Environmental systems research institute; FRM: Federal reference method; HB: Hierarchical Bayesian; IQR: Interquartile range; KM: Kilometer; MCAPS: Medicare air pollution study; NAAQS: National ambient air quality standards; PM: Particulate matter; RD: Relative difference; SAS: Statistical analysis system; U.S.: United states

## Competing interests

The authors declare that they have no competing interests.

## Authors’ contributions

AV acquired the data, analyzed and interpreted the data, contributed to design of the study, and drafted and revised the manuscript. WFD contributed to acquiring the data, interpretation of data, and design of the study and drafted parts of the manuscript and made revisions to the manuscript. SRK made contributions to the interpretation of data, and drafted parts of the manuscript and made critical revisions to the manuscript. JRQ made contributions to design and revision of the manuscript. All authors read and approved the final manuscript.
